# Evolutionary Biology Needs Wild Microbiomes

**DOI:** 10.3389/fmicb.2017.00725

**Published:** 2017-04-25

**Authors:** Sarah M. Hird

**Affiliations:** Department of Molecular and Cell Biology, University of ConnecticutStorrs, CT, USA

**Keywords:** gut microbiome, field biology, evolution, ornithology, host-associated microbiota

## Abstract

The microbiome is a vital component to the evolution of a host and much of what we know about the microbiome derives from studies on humans and captive animals. But captivity alters the microbiome and mammals have unique biological adaptations that affect their microbiomes (e.g., milk). Birds represent over 30% of known tetrapod diversity and possess their own suite of adaptations relevant to the microbiome. In a previous study, we showed that 59 species of birds displayed immense variation in their microbiomes and host (bird) taxonomy and ecology were most correlated with the gut microbiome. In this Frontiers Focused Review, I put those results in a broader context by discussing how collecting and analyzing wild microbiomes contributes to the main goals of evolutionary biology and the specific ways that birds are unique microbial hosts. Finally, I outline some of the methodological considerations for adding microbiome sampling to the research of wild animals and urge researchers to do so. To truly understand the evolution of a host, we need to understand the millions of microorganisms that inhabit it as well: evolutionary biology needs wild microbiomes.

## Introduction

Animals evolved in a microbial world: prokaryotes precede animals by ~3 billion years (Hickman, 2005). Thus, it may not be surprising that every animal is a **host** for a complex microbial community (its **microbiome**), containing billions to trillions of microorganisms, belonging to hundreds to thousands of species (Bäckhed et al., 2005; Qin et al., 2010; Human Microbiome Project Consortium, 2012) from all divisions of life (Bacteria, Archaea, and Eukaryotes), as well as viruses. There are an estimated near equal number of microbial and human cells on the human body (Sender et al., 2016) and microbial genes may outnumber a host's genes by orders of magnitude (Hooper and Gordon, 2001; Qin et al., 2010).

KEY CONCEPT 1 **Host**

A “host” is any living thing that houses a microbial community, although the term can be applied broadly (like discussing “the avian microbiome”) or specifically (“the microbiome of the flight feathers of pet parakeets”).

KEY CONCEPT 2 **Microbiome**

“Microbiome” refers to the collective microorganisms living in a particular environment; qualifying terms, like “human microbiome” or “chicken fecal microbiome” specify the microorganisms living in particular environments.

The microbiome is of fundamental importance to vertebrates. In addition to aiding digestion (Hooper et al., 1998) and facilitating energy extraction and storage (Bäckhed et al., 2004; Turnbaugh et al., 2006), it is involved in growth and organ development (Diaz Heijtz et al., 2011; Erny et al., 2015), immune system maturation (Mazmanian et al., 2005; Chung et al., 2012), behavior (Dinan et al., 2015), and defense against pathogens (van der Waaij, 1989). The microbiome can affect mate choice (Sharon et al., 2010) and mating success (Brucker and Bordenstein, 2013), directly linking the microbiome and host evolution.

Most microbiome research to date has been on humans and model organisms. But captivity alters the microbiome in mammals (Uenishi et al., 2007; Delsuc et al., 2014; Kreisinger et al., 2014; Clayton et al., 2016; Delport et al., 2016), birds (Scupham et al., 2008; Matsui et al., 2010; Wienemann et al., 2011; Rodríguez-Ruano et al., 2015; Wang et al., 2016), fish (Dhanasiri et al., 2010), reptiles (Keenan et al., 2013), and amphibians (Loudon et al., 2013; Becker et al., 2014; Bataille et al., 2016), which is likely due to the dietary, social, and environmental conditions of captivity that are so different from those experienced in the wild. Captive microbiomes likely do not represent the natural variation of the microbiome of a species (or population), which is necessary for evolutionary analysis.

Microbiomes are a relatively new frontier in evolutionary biology but this is not because their existence was unknown or thought unimportant. Instead, recent methodological advances now allow researchers to sequence the DNA of members of these communities without culturing each organism first and we can do so at a reasonable cost. In this Frontiers Focused Review, I discuss how sampling the microbiomes of wild organisms contributes to the goals of evolutionary biology. I then highlight the ways in which birds are unique and important microbial hosts and outline methodological considerations for adding microbiome research to field studies. Now that we have the tools, it is time to acknowledge and explore the fundamental importance of microbiomes in the evolution of hosts and to include them in field studies when possible.

## The goals of evolutionary biology

**Figure 1:**
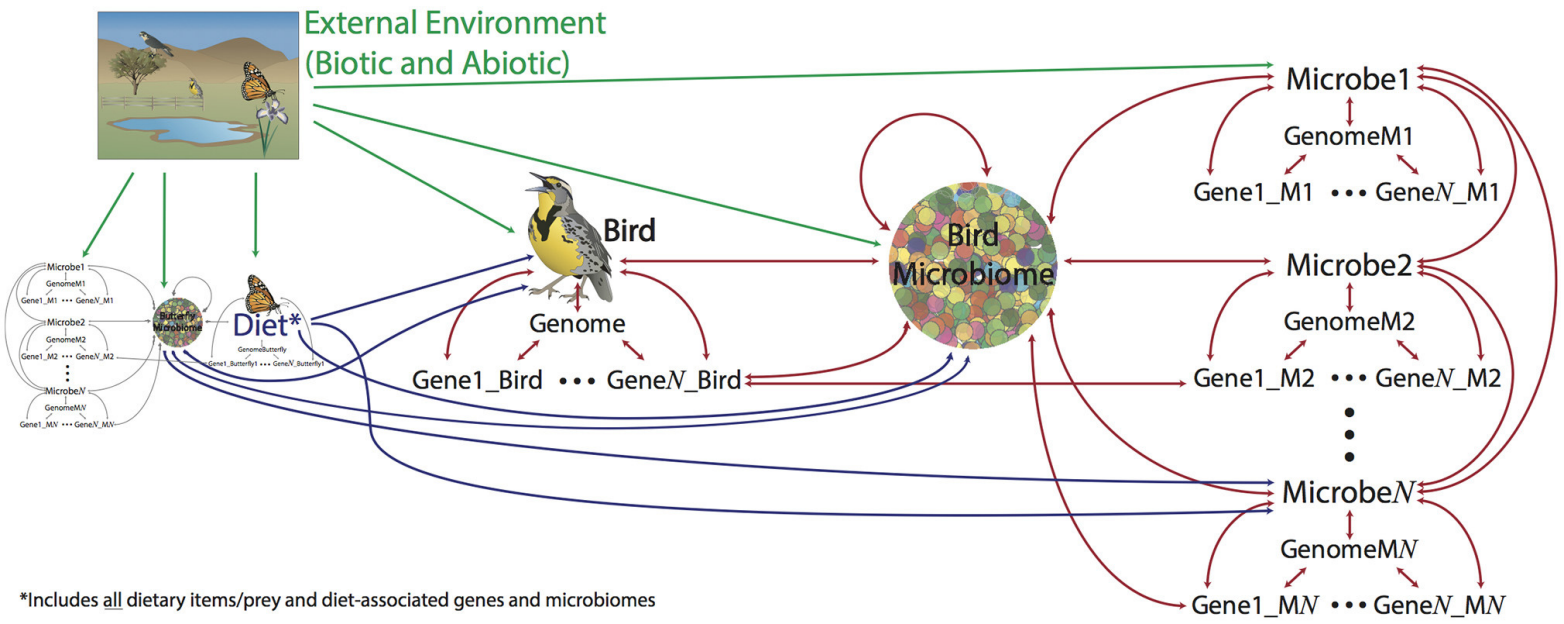
**The goals of evolutionary biology are to describe and understand living things, including their distribution, lifestyles, and history**. Without microbiomes, we are missing not only the majority of living things on the planet (bacteria) but also important interactions between dynamic forces. Arrows on figure show how different levels of biological organization can affect each other. E.g., eating a butterfly affects (in a broad sense) a bird; the microbiome of the butterfly can also affect the bird, as well as directly affect the microbiome of the bird. Genes and genomes of all living pieces of this “foodweb” interact at many scales and the evolution of all the pieces are connected to many others.

Who shares our planet and where do they live? How and when did they get there? What are they doing, what role do they play in their communities? These general questions fall under three main goals of evolutionary biology: To (1) discover and describe biodiversity, including estimating phylogeny, (2) understand the natural history and lifestyle of an organism (or group), (3) elucidate the forces responsible for the natural history and phylogeny of an organism (or group). Microbiomes present a unique opportunity for understanding evolution as they are both a force whose emergent properties affect a host, but also a community of millions or more individuals, each with their own genomes and evolutionary history. They complete the network of biological interactions between “individuals”—genes, microbes, and hosts—and “communities”—genomes, microbiomes, communities—where every level can influence the others (Figure 1). With modern technologies we can synthesize genomic, ecological, and environmental data into a more complete understanding of biodiversity and evolution.

### Goal 1: discover and describe biodiversity, including phylogeny

Somewhere between several million (Schloss et al., 2016) and one trillion (Locey and Lennon, 2016) bacterial species inhabit the earth and most of these are not accessible through culture-based techniques (Handelsman, 2004). As the first aim of evolutionary biology (Table 1) is to document life on earth, microbiome research advances this goal by identifying organisms that are otherwise invisible to science (Wright et al., 2009). This is especially true since novel habitats uncover novel biodiversity (e.g., Goffredi et al., 2008; Goffredi, 2010; Petersen et al., 2010; Hug et al., 2016). For example, hoatzins are the only folivorous, foregut-fermenting bird and their crop and lower esophagus function as an extended fermentation chamber for the leaves in their diet. Hoatzin crop microbiomes contain many novel bacterial strains: one study including six wild individuals found 94% of the bacterial **OTUs** belonged to unnamed species (Godoy-Vitorino et al., 2008). Another study, focusing on Archaea, found 17 of 24 methanogen OTUs likely represented new species and perhaps three new genera (Wright et al., 2009).

**Table 1:** **The three main goals of evolutionary biology, applied to the microbiome (MB)**.

**Discover and describe**	**History and lifestyle**	**Processes and forces**
What is here? (including: What lives here? What's passing through? What's living? What dead?)	Where does the MB come from? How is the MB seeded (from where)? (Metacommunity dynamics)	Roles of different layers of selection? (on host, on MB, on microbe, on genomes, on genes)
What always lives here? (“core microbiome”)	How does the MB change over time? (succession)	Role of social contact between hosts (microbial migration)
Why is it here? (Resident or transient; living; or dead)	Analysis of phylogeny of microbes within host (including concepts of adaptive radiation, HGT, gut biogeography)	Role of pathogens (disturbed state, succession dynamics, or source-sink dynamics); role of disease or illness of host on MB
How many unique taxa? What is new to science? (endemism)	Analysis of phylogeny of particular microbes across hosts (including concepts from phylogeography)	(How) Does MB aid adaptability of host? (How) Does the MB adapt to new environments?
Estimate phylogeny of bacteria within a host; estimate phylogeny of bacteria (or clade) across hosts	What is the maternal contribution (vertical and pseudo-vertical inheritance)?	Testing for, identifying, quantifying coevolution between host and microbe(s). Differentiating from co-diversification
Defining a bacterial species, a pan-genome	What is the neonatal environment's contribution?	What is the community structure?
Which taxa co-occur?	What is host ecology's contribution (e.g., diet)?	Extinction/speciation: of microbe and host

KEY CONCEPT 3 **OTU**

OTU stands for “operational taxonomic unit”—a broad term that is used to classify organisms of unknown taxonomy into groups. OTUs are frequently defined by how similar DNA sequences are (e.g., “99% OTUs” group all sequences within 99% similarity into a single OTU) and conceptually similar to the concept of a species.

### Goal 2: understand natural history, lifestyle, and traits

The second goal of evolutionary biology is to understand the lifestyle and history of an organism. Here we want to know *what* an organism does, *where* it does it and *when* it existed (Table 1). Describing traits such as home range, species range, daily and seasonal habits, physiological adaptations, phylogeography, etc., are essential for understanding how an organism functions in the world.

Vultures provide an excellent example of how microbiomes add biological value to our understanding of host traits. Vultures are carrion feeders and this dietary specialization exposes them to many microbes that are known pathogens. Roggenbuck et al. (2014) investigated whether the microbiome enables this lifestyle by providing resistance to pathogens. Black vulture and turkey vulture contain high bacterial diversity on their head skin and extremely low bacterial diversity in their gut. Quite unusually, the bacterial classes *Fusobacteria* and *Clostridia* dominate and their relative abundances are similar across species and in a captive vulture as well. *Fusobacteria* and *Clostridia*, which are frequently pathogenic to other bird species, are possibly contributing to carrion digestion in the vulture digestive tract and outcompeting other bacteria that may be suitable for the niche. The avian hindgut is a previously undescribed niche for both bacterial taxa. Thus, *Fusobacteria* and *Clostridia* may facilitate the vulture lifestyle.

There are many available methods to assess the microbiome as a trait. Phylogenies are one powerful tool that provide evolutionary information about the microbiome. Phylogenies can establish which bacteria are important to host or biogeographic micro-environment and can be used to calculate descriptive (alpha diversity) and comparative (beta diversity) statistics. Phylogenetic distance metrics like UniFrac (Lozupone and Knight, 2005) can determine how similar microbial communities are and permutation tests assess significance. Bacterial phylogenies can indicate how long a microbial taxon has inhabited a particular environment (Moodley et al., 2012; Moeller et al., 2016). **Categorical statistical tests** are another tool that correlate host traits with microbiomes (Godoy-Vitorino et al., 2012; Hird et al., 2014, 2015). Functional analysis of microbiomes using **metagenomics** can illuminate what genes and pathways are found in a sample. Microbiome functions may be more conserved than taxonomic composition (Lozupone et al., 2012), since convergence, horizontal gene transfer, and functional redundancy may select for function rather than taxonomy. When coupled with host genomic information, a powerful method to detect concurrent shifts in host and microbial function emerges (Gao et al., 2016).

KEY CONCEPT 4 **Categorical statistical tests**

“Categorical statistical test” is a broad term meaning any statistical test that can detect a correlation between metadata associated with a sample and the microbial community. Examples include Anosim, Adonis, MRPP, Permanova.

KEY CONCEPT 5 **Metagenomics**

Metagenomics is a group of methods that uses pieces of random genes from a mixed microbial sample to infer the gene content of the sample. Shotgun metagenomics sequence random DNA fragments directly. Functional metagenomics express genes in a bacterial host organism and screen the cellular activities to ascribe gene function. Metatranscriptomics analyze RNA instead of DNA to identify genes that are actively being transcribed in a sample.

### Goal 3: elucidate forces and processes affecting natural history and phylogeny

The third major goal of evolutionary biology moves from describing natural history and phylogeny into elucidating the processes that shaped them. All of the major evolutionary forces—selection, drift, migration, mutation, diversification, extinction, and adaptation—are as fundamental to the microbiome are they are to hosts yet at this point we have more questions than answers (Table 1). The microbiome is under multiple levels of selection (Ley et al., 2006): how strong are the different levels and how do they structure the microbiome? Drift (including founder effects and bottlenecks) may be important during times of illness or early in life, when microbial populations are small. How genetic drift in the host affects the microbiome is unknown. The (relatively) rapid generation time of microbes and the ability of the community to change in response to stimuli may contribute to acclimation in hosts, which in turn could facilitate adaptation of a host to new or changing environments (Alberdi et al., 2016). The microbiome facilitated the mammalian expansion from carnivory to herbivory (Ley et al., 2008) and it contributes to more minor ecological shifts as well (like the ability to consume toxic plants; Kohl et al., 2014, 2016). Host migration and population structure may drive the extinction of distinct microbial taxa (Domínguez-Bello et al., 2008).

**Figure 2:**
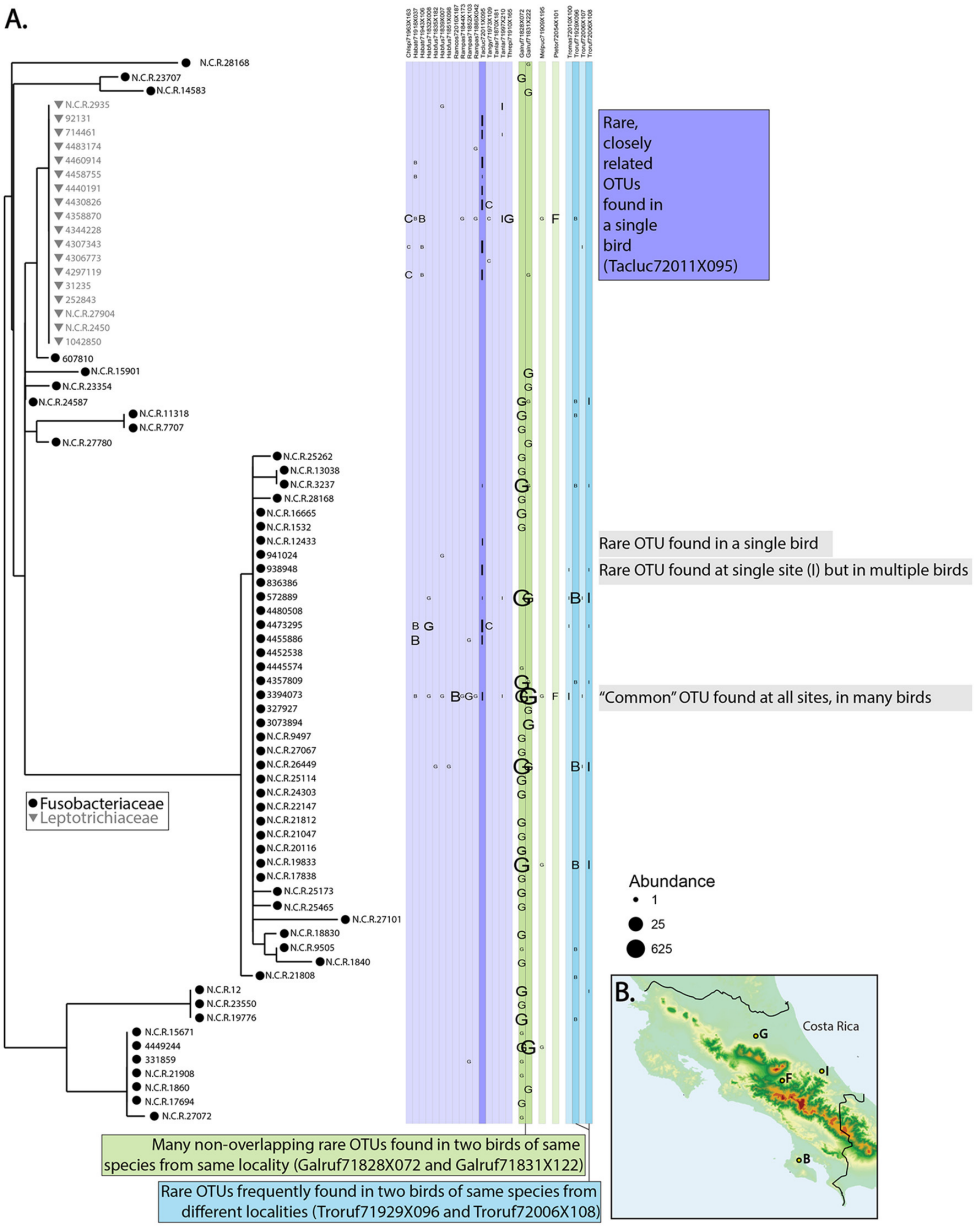
**Relationship between bacteria and birds. (A)** Phylogenetic tree of bacteria belonging to the order *Fusobacteriales* and which hosts the bacteria were found in. All members in the original dataset belonging to five bird families are shown for comparative purposes; bird orders are grouped by color and the first six letters of each name represent the species (see Hird et al., 2015, for more information about samples). Whether a particular OTU was found in a particular bird is shown in columns where the letter denotes which sampling locality the bird came from (on map shown in **B**) and the size of the letter refers to the abundance of the OTU. Patterns of note are shown on the figure.

Comparing microbiomes in the wild contributes to all three of the major goals of evolutionary biology. Hird et al. (2015) generated a biodiversity catalog for the microbiomes of 59 Neotropical bird species and improved our understanding of both birds and microbiomes in an evolutionary context. First, since Hird et al. (2015) contained the first microbiome information from any of the host species, we described new environments for every microbe identified, thus contributing to Goal 1 of evolutionary biology (although whether the OTUs are residents or transients, living or dead, is unknown). The Hird et al. (2015) data also contribute to Goal 2: the microbiome—as a trait—has been described for the host species and revealed some interesting patterns. For example, Figure 2 shows the distribution of Fusobacteriales across a subset of the birds in Hird et al. (2015) and the distribution of the bacteria gives some information about the hosts. Some birds appear to be better suited for hosting *Fusobacteriales*—the two samples in dark green belong to two *Galbula ruficauda* (Galruf71828X072 and Galruf71831X222). They have many more *Fusobacteriales* than all other birds, including those from the same family (shown in lighter green) and those from the same sampling locality (site G). This supports the hypothesis that *G. ruficauda* may be selecting for *Fusobacteriales* in their gut; on a phylogenetic tree, many short branches from a single environment may indicate adaptive radiations (Ley et al., 2006), an intriguing possibility for *G. ruficauda*. Furthermore, the OTUs found in GALRUF71828X072 and GALRUF71831X222 are abundant and closely related, possibly indicating diversification of *Fusobacteriales* within the species, *G. ruficauda* or the individuals themselves.

## Birds as microbial hosts

Modern birds are a globally distributed (Jenkins et al., 2013), economically and socially important, ecologically, and morphologically diverse clade that began diversifying in the mid-Cretaceous, around 95–115 million years ago (Lee et al., 2014). They are ubiquitous. We know very little about how the microbiome influences avian hosts, especially in the wild. Approximately 90% of published microbiome research has been on mammals (Colston and Jackson, 2016), but mammals and birds are different in many ways that likely influence the microbiome. For example, mammals are birthed through a microbially rich vaginal canal that directly shapes the microbiome (Dominguez-Bello et al., 2010). Mammals are fed milk produced by the mother that dynamically responds to the needs of the baby (Hassiotou and Geddes, 2015).

A bird, on the other hand, lays fertilized eggs into a nest, which is a highly variable structure (Mainwaring et al., 2014). Nest building alters the feather microbiome (Saag et al., 2011; Kilgas et al., 2012) and over time, nest and maternal microbiomes converge (Goodenough et al., 2017). Nests frequently include materials with antimicrobial properties, such feathers (Peralta-Sánchez et al., 2010) and plants (Dubiec et al., 2013; Mainwaring et al., 2014; Ruiz-Castellano et al., 2016). Nest construction behavior, like nest or nesting material reuse or removal, may also affect the microbial ecology of the nest (González-Braojos et al., 2012). How does this built environment affect the host microbiome?

The eggs leave the mother through her cloaca, the bird's joint terminus for the excretory, urinary, and reproductive system that is in contact with the outside environment and mates. Microbes derived from each of these systems may be present in the cloaca. A parent then incubates the egg until it hatches, a behavior that can affect egg microbial load (Cook et al., 2005a,b; Shawkey et al., 2009). Some birds excrete an antimicrobial substance from their uropygial gland and physically cover the eggs with it (Soler et al., 2014; Martínez-García et al., 2016); eggs can even be physically suited to retain the uropygial oil (Martín-Vivaldi et al., 2014). Babies are then fed from the mouth/crop of a parent or if they are precocial, may start feeding themselves immediately. The effects of the initial food source are likely important and deserve further study; what is the influence of the early environment on bird microbiomes?

The ability to fly put selection pressure on avian digestive efficiency, resulting in short retention times of food, and exaggerated differences in gut morphology (Stevens and Hume, 1995). How the speed of digestion and plasticity of the alimentary canal affects the microbiome in birds is relatively unknown. Feathers are an ancient structure unique to birds that facilitate flight and are crucial to avian health and wellness. Significant energy goes into growing and maintaining feathers (Walther and Clayton, 2005), which protect the bird from the elements, predators and parasites, as well as attract mates. The main function of the uropygial gland is to produce an oily, protective substance that is applied to feathers during preening. Uropygial secretions contain antimicrobials (Soler et al., 2012; Czirják et al., 2013) that can be effective against feather degrading bacteria (Ruiz-Rodriguez et al., 2009) and can respond to changes in environmental bacterial load (Jacob et al., 2014; Leclaire et al., 2014). Furthermore, all birds lose and regrow their feathers at least once a year through molting, a physiologically expensive process that can alter the flight and feeding patterns of birds. Molting is associated with changes in the fecal microbiome in two species of penguin (Dewar et al., 2014).

Finally, social contact is correlated to the microbiome in birds (Møller et al., 2009; Levin et al., 2016). Birds display a wide variety of social behaviors, varying from a largely solitary lifestyle (like hummingbirds) to a highly gregarious one (like many blackbirds). Parental care is also highly variable; some species share parental duties from incubation to fledging and others provide no parental care at all (like brood parasites). Sexual contact appears to affect the cloacal microbiome: repeated contact between sexual partners homogenizes the cloacal microbiome of barn swallows (Kreisinger et al., 2015) and sexual contact changes the female cloacal microbiome in kittiwakes which reverts to pre-copulatory status as time since intercourse increases (White et al., 2010). The extent to which sexually transmitted microbes affect the microbiome of other biogeographic sites is unknown.

To better understand birds and their unique features, we need to incorporate their microbiomes into ornithological and evolutionary biology research. We need to characterize microbiome diversity at the population level, as well as across all branches of the Avian tree of life. We need to determine the salient environmental and morphological metadata to describe and compare the microbial samples. Wild microbiomes are necessary to complete these tasks and to transition from descriptive to explanatory research. Approximately one in eight bird species is threatened with extinction (Bird Life International, 2015); understanding microbiomes may help prevent extinction, both of birds (by informing animal husbandry, management or conservation priorities) and endemic microbes. Collection of microbiome data should be a priority for future field studies.

## Field collection of wild microbiomes

Field collection of microbiomes offers unique challenges that are briefly discussed below. General advice on conducting microbiome studies has been reviewed elsewhere (Kuczynski et al., 2011; Goodrich et al., 2014).

### Study design

**Figure 3:**
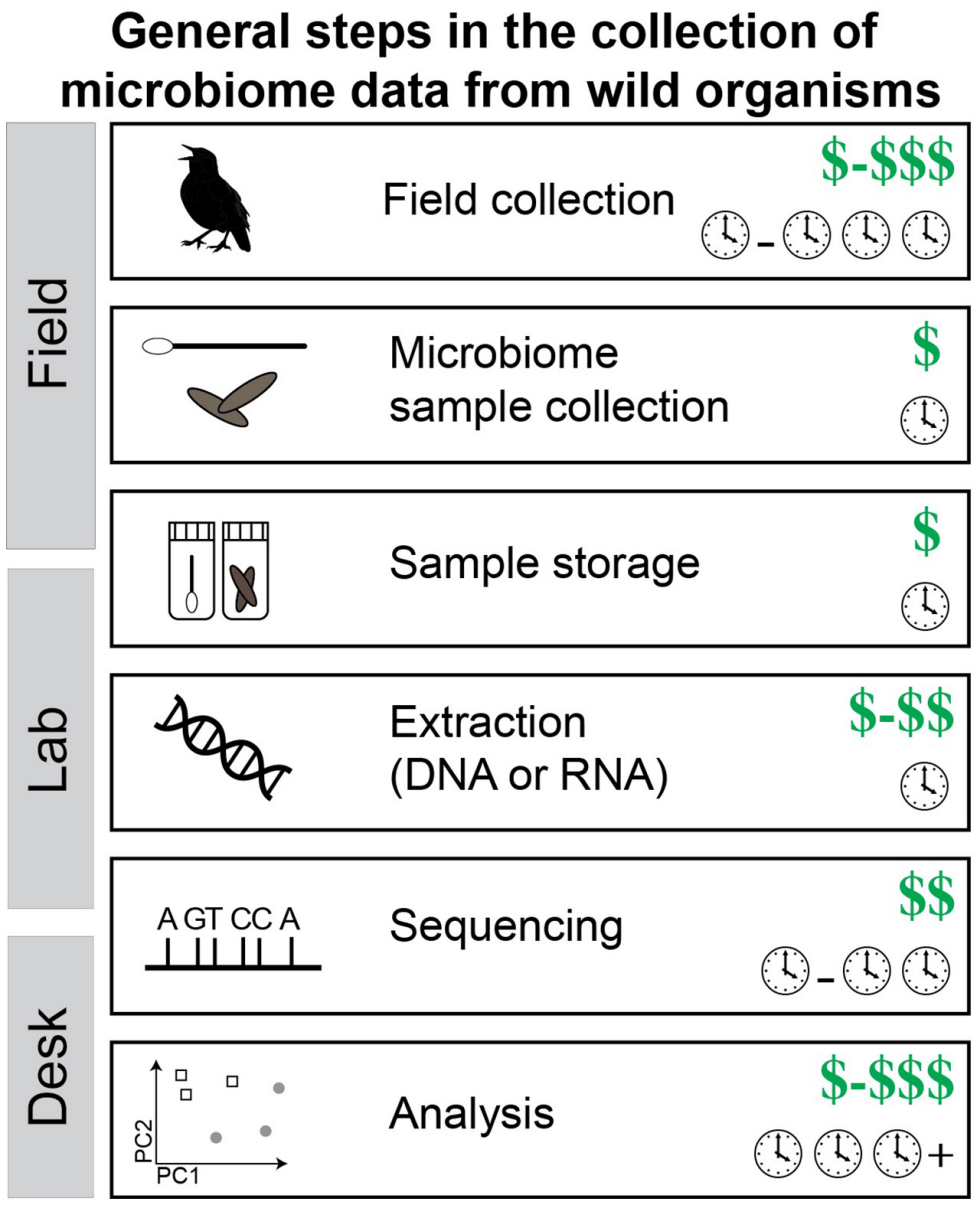
**The steps required to collect microbiome data from wild organisms**. Note that the cost of each step is shown: dollar signs represent cost of raw materials and clocks represent time investment. Values shown are estimates of the expected minimum cost but can vary, sometimes by quite a bit. Notably, time equals money in many cases (e.g., personnel).

Study design is of utmost importance for field microbiome studies because increasing sample size at a later date may be difficult to impossible. Additionally, microbiomes can change over time or with season (Bailey et al., 2010; Liang et al., 2015), so securing appropriate sample size during a field trip or field season is best. The research question will largely drive the sampling but the desired molecular data can greatly influence the budget. There is a direct tradeoff between number of samples one can analyze and the sequencing coverage per sample and this equation needs to be carefully considered. Collecting **replicate samples** is essential (if possible) because the variation within species (or groups) is likely unknown and the results from a single sample may be misleading. Microbiome samples are relatively cheap to collect in both time and money (Figure 3). This is especially true in comparison to the cost of getting to some field locations or effort to get the host organism in hand.

KEY CONCEPT 6 **Replicate samples**

Replicate samples are multiple samples from the same host species, locality, time point, disease state, etc. Individual variation can be very high between microbiome samples, so getting multiple samples within the categories of interest is imperative to distinguish signal from noise and error.

### In the field: sample preparation and storage

Sample preparation and storage of microbiomes is very important: decomposition begins within minutes of death (Vass, 2001) and triggers a successional change in the microbiome (Metcalf et al., 2016). Bacterial taxa in feces can change after <30 min at room temperature (Gorzelak et al., 2015). Freezing, with or without buffer, is thought of as a “gold standard” for sample preservation (Song et al., 2016). This is plausible for some field trips, if access to a freezer, dry ice or liquid nitrogen is available. However, field conditions often prohibit immediate freezing of the sample. High purity ethanol (95% or higher) can fix the bacteria in a sample and this method has been shown to work especially well with fecal samples; Hale et al. (2015) found that frozen and ethanol stored samples had the most similar microbial communities to fresh fecal samples after 8 weeks of storage. Liquid storage buffers are another field possibility and can preserve both RNA (e.g., RNAlater) and DNA [DNAgard or non-proprietary solutions like DMSO/EDTA/saturated sodium chloride (DESS)]. Consistency may be the most important factor for field storage of wild microbiome samples—several storage methods perform well but comparing across methods may be problematic.

### Molecular methods

As the costs involved in sequencing will affect sampling design, it is best to have a plan for the molecular work from the beginning of a study. The two main categories of microbial community genetic data are amplicon-based and metagenomics. **Amplicon-based studies** are those that amplify and sequence a single homologous locus (usually a variable region of **16S rRNA**). These studies are an informative and economical first step in describing and analyzing microbiomes. The data provide taxonomic information (using large, publicly available databases) and diversity statistics that can be used to characterize and compare microbial communities. Amplicon-based studies are limited, though, in that they (1) rely on PCR, (2) produce short sequencing fragments, (3) contain only one marker. In many cases, information about the functional capabilities of a community are of interest. Functional information can be estimated from 16S rRNA data with computational approaches (Langille et al., 2013) but how well they perform in novel environments is unknown.

KEY CONCEPT 7 **Amplicon-based studies**

Amplicon-based studies use PCR to amplify a single genetic locus prior to sequencing. All data can be phylogenetically compared because they are homologous. The variable regions of the 16S rRNA gene are the most popular example of amplicon-based studies.

KEY CONCEPT 8 **16S rRNA**

The 16S rRNA gene is a popular choice for amplicon-based studies because it contains highly variable regions (suitable for comparative analysis) flanked by highly conserved regions (suitable for placing PCR primers). Although PCR may bias results and even the “universal” primers may miss some diversity, it is a popular option for comparing microbiomes (especially including consideration of cost).

Alternative to amplicon-based methods are shotgun metagenomics methods, which analyze a random subset of genetic material from a sample (Zarraonaindia et al., 2013). One shotgun metagenomics approach is sequence-based metagenomics. Here, random DNA fragments from microbial communities are sequenced and used to infer the genes or metabolic pathways found in a sample based on available databases, e.g., KEGG Orthology (Kanehisa et al., 2004). With sufficient coverage and analytical tools, full or nearly complete genomes can be reconstructed from a metagenome (Tyson et al., 2004; reviewed in Sangwan et al., 2016). Another shotgun approach, functional metagenomics, can be used to screen genomic fragments for specific traits (e.g., antibiotic resistance). Here, fragmented DNA is cloned into a fosmid vector then transfected into a bacterial host (frequently *E. coli*), where functional experiments can be performed (e.g., Sommer et al., 2009). Metagenomics are generally more expensive than amplicon based studies, as they require greater sequence coverage to describe a sample and construct high quality contigs, and require more powerful computational tools to analyze. Metatranscriptomics is another option that uses the RNA in a sample to determine what genes are actively being transcribed at the time of sampling (e.g., Franzosa et al., 2014). Like metagenomics, this method can be expensive but is informative about the metabolic processes occurring at a given time.

### Analysis

The variable regions of the 16S rRNA molecule are by far the most popular choice for characterization of a microbiome. There are many software packages that are suitable for microbiome sequence analysis, the most popular of which are free and include extensive tutorials [e.g., QIIME (Caporaso et al., 2010), mothur (Schloss et al., 2009)]. Metagenomic and metatranscriptomic analyses are more involved than single locus analyses, requiring contig assembly before annotation and alignment to databases. Oulas et al. (2015) provides a detailed description about the computational steps involved in analyzing metagenomic data. All bioinformatics analyses require computational resources; these can range from relatively cheap, e.g., if one has access to an institutional cluster to extremely expensive e.g., if one needs to purchase hardware or cluster time. Additionally, analysis is frequently the most time-expensive step, which directly translates to dollars in many instances. Many sequencing facilities can add standard bioinformatics analysis to a project for a fee.

## Conclusions

The microbiome is important to its host in many ways and investigating this relationship in an evolutionary context is both possible and imperative. Through the microbiome we learn about individuals and communities, hosts and microbes, genes, and genomes. These investigations achieve the three main goals of evolutionary biology—to discover the Earth's biodiversity, understand its history and illuminate the forces that generated it. Preserving microbiomes as a routine part of evolutionary studies adds an important component to the web of biological interactions and generates questions for continued exploration. Wild microbiomes provide important data to evolutionary biology, but only if we look.

## Author contributions

The author confirms being the sole contributor of this work and approved it for publication.

## Funding

The author wishes to thank the University of Connecticut and the University of California Davis Chancellor's Post-doctoral Fellowship for funding.

### Conflict of interest statement

The author declares that the research was conducted in the absence of any commercial or financial relationships that could be construed as a potential conflict of interest.
